# A High-Performance Day-Age Classification and Detection Model for Chick Based on Attention Encoder and Convolutional Neural Network

**DOI:** 10.3390/ani12182425

**Published:** 2022-09-15

**Authors:** Yufei Ren, Yikang Huang, Yichen Wang, Shijie Zhang, Hao Qu, Jie Ma, Longhe Wang, Lin Li

**Affiliations:** 1 College of Information and Electrical Engineering, China Agricultural University, Beijing 100083, China; 2 Institute of Animal Science, Guangdong Academy of Agricultural Sciences, Guangzhou 510640, China; 3 National Research Facility for Phenotypic and Genotypic Analysis of Model Animals (Beijing), Beijing 100083, China

**Keywords:** chick day-age classification, precision livestock, convolutional neural network, edge computation, attention encoder, artificial intelligence application

## Abstract

**Simple Summary:**

Most management methods for poultry farming currently rely on human labor. Such management is labor-intensive and inefficient, especially in identifying poultry growth stages. Given the lack of a high-precision artificial method for chick’s day-age detection, a high-performance day-age classification and detection model for chicks was proposed based on artificial intelligent techniques. This method can detect and classify chicks in six different living stages from 1 to 32 day-ages, and the accuracy is 95.2%, superior to other current ones. In order to apply this method in practical scenarios, it has been deployed into an application based on the IOS system, which can recoganize the day-age of chicks by capturing real-time photos. The system is currently deployed in Rizhao City, Shandong Province, China. It helps chicken farm staff automatically detect the behavior of chickens, whose excellent working effect proves the robust availibility of the proposed method.

**Abstract:**

Thanks to the boom of computer vision techniques and artificial intelligence algorithms, it is more available to achieve artificial rearing for animals in real production scenarios. Improving the accuracy of chicken day-age detection is one of the instances, which is of great importance for chicken rearing. To solve this problem, we proposed an attention encoder structure to extract chicken image features, trying to improve the detection accuracy. To cope with the imbalance of the dataset, various data enhancement schemes such as Cutout, CutMix, and MixUp were proposed to verify the effectiveness of the proposed attention encoder. This paper put the structure into various mainstream CNN networks for comparison and multiple ablation experiments. The final experimental results show that by applying the attention encoder structure, ResNet-50 can improve the accuracy of chicken age detection to 95.2%. Finally, this paper also designed a complete image acquisition system for chicken houses and a detection application configured for mobile devices.

## 1. Introduction

The day-age of a chicken is a concept similar to age calculated in years, while the day-age of a chicken is counted in days. The day-age of chickens is an extremely important indicator in chicken breeding and production. Chickens of different days have large differences in their physiological state. Chickens of different physiological states require different feeding management conditions. Divide the stages according to the physiological development of chickens to facilitate targeted feeding management, which is conducive to the healthy growth of chickens and improves their production performance. The general growth and development stages of chickens [1] are divided according to their day age:

1. Chicks: from newborn to 60 days old;
2. Child chickens: from 61 to 150 day-ages for the egg type and up to 180 day-age for the part-time type;
3. Reserve chickens: new chickens that have not yet started laying eggs or breeding roosters that have not been bred.

Raising reserve chickens well is a stage that cannot be ignored to improve the production and breeding value of the chicken population. In terms of different classification standards, including production status, type of production, and development stages, the detailed category information of chickens is displayed in Table 1.

By calculating the age of chickens and classifying chickens into stages, the following production arrangements can be effectively carried out. These measures contain:

1. Predicting and improving the new breeding environment.
2. Determining whether chickens have reached the appropriate breeding, fertility, or food standards for breeding scale expansion.
3. Predicting the susceptibility of chickens to diseases such as abdominal fat deposition [2] and bone quality problems [3] at different times and applying appropriate control measures, etc.

To determine the day-age of chickens, the following methods are usually used: First, look at the beak nail. Young chickens have sharp and thin beak nails, narrow and thin beak corners on both sides, and no crusts. Chickens over a year old have slight crusts. In contrast, adult chickens have thick and short beak nails with hard and slippery ends, broad and rough corners on both sides of the beak, and large crusts. The second way is to look at the nasal tumor. The nasal tumor of mammary chickens is red, the nasal tumor of child chickens is light red and shiny, the nasal tumor of chickens over two years old has a light pink color, and the nasal tumor is larger and soft, moist and shiny. The nasal tumors of chickens over four or five years old are pink and rougher. The third way is to look at the toes. Young chicken feet are fine and soft. They have soft, flat, tiny scales on their feet and inconspicuous scale patterns. Their feet are bright red, with soft and pointed toenails and a soft texture. Adult chicken feet are stout, with thick and hard scales, clear scales, dark red, and hard and curved toenails. The fourth method is to look at the feathers. The main wings of the chicken can be used to identify the month-age of child chickens [4]. These commonly used ways, however, can only roughly classify chickens and cannot be pinpointed to their specific day-age. If we want to record the day-age of chickens, we need to be pinpointed from the day they are born, marked by physical methods of establishment, and then recorded by manual methods. Alternatively, physical division of chickens of the same day-age and keeping chickens of different day-ages in different locations for differentiation would be labor-intensive. It is highly costly in terms of labor and requires a large space to create a physical barrier for the chickens. At the same time, accuracy can not be guaranteed, and there are always some human errors that cannot be quantified.

Therefore, it is essential to use artificial intelligence to determine the chickens’ day-ages. Improving the accuracy and precision of chicken day-age recognition has many positive implications for chicken growth and reproduction. Firstly, the susceptibility of chickens to diseases can be predicted by day-age, and effective treatment or prevention can be carried out in advance, including but not limited to isolation and the use of drugs to effectively prevent the spread and deterioration of the disease [5]. Secondly, during different periods of egg growth, it is necessary to use scientific methods to carry out reasonable egg breeding and to separate pens for malnourished hens to ensure their daily energy intake. During the brooding and breeding period, it is important to ensure sufficient vitamin and amino acid [6] intake for the chickens’ development and maturity. During the high laying period, it is crucial to provide sufficient protein and calcium to prolong the duration of peak egg production and thus increase egg production [7]. The organic acid is also an important indicator [8]. By judging it, nutrition can be increased for chickens in time to improve egg-laying efficiency. Thirdly, through day-age judgment, chickens with the best export taste and different meat quality can be precisely selected for sale. It can improve the quality of chicken meat in catering and provide different chicken meat for different dishes and customer needs, improving meat quality in general [9]. Fourth, the day-age can be used to determine the preferences and interests of chickens, and then the living environment conditions can be changed and updated, which is conducive to improving animal welfare, which is judged by animal behavior [10].

Taxonomists have been searching for more efficient methods to meet species identification requirements, such as developing digital image processing and pattern recognition techniques [11], using camera traps for objection detection, which would identify species accurately and concisely. Object recognition in images is a relatively new field and the first (deep) convolutional neural network architecture and early achievement in text document recognition was described by LeCun in 1998 [12]. In 2007, Szabolcs Sergyán implemented an image classification based on color content, where the colors are stored in the intensity vector of image pixels, and the information can be easily retrieved. Colors can be represented in different color spaces or features and can be stored in several ways. RGB is a color space widely used for image display [13].

Face recognition has already been a widely used technology, and it has been applied in many areas. Graham, E.H. et al. proposed a new GoogLeNet-M network with regularization and migration learning. It is demonstrated that the regularized GoogLeNet-M network with migration learning has the best performance, with a recall of 0.97 and an accuracy of 0.98. [14]. Fang, Cheng et al. used a deep neural network technique to estimate the chicken pose. The standard deviation of the accuracy of the method proposed in this paper is 0.0128, and the confidence level (95%) is 0.9218 ± 0.0048. The other case is the standard deviation of recall is 0.0266, and the confidence level (95%) is 0.899 6 ± 0.0099 [15].

After achieving significant results in face recognition, developments also started in pig face recognition. The adaptive pig face recognition method based on the convolutional neural network developed by Marsot achieved 83% accuracy, promoting the application of artificial intelligence for animal recognition in pig production [16]. Li, G. et al. designed a noninvasive pig face recognition method based on the improved YOLOv3 recognition method. This method allows multiple pigs to be recognized simultaneously. The results show that the YOLOv3_DB_SPP model improves the feature extraction ability of the primary feature extractor and the accuracy of the detector [17]. Li, S. et al. further proposed a method for individual pig recognition based on the improved YOLOv4 convolutional neural network. The results showed that the test set’s average accuracy (mAP) reached 98.12% when the threshold value was 0.5. The recall rate reached 95%, the F1 score was 96%, the average recognition time Mean FPS was 34.3ms, and the average crossover ratio (IoU) was 83.91%. Compared with the improved model of Fast R-CNN, YOLOv3 improves the recognition accuracy and speed of individual pigs; meanwhile, the recognition accuracy of the pig body dataset is significantly improved compared with the traditional pig face dataset [18].

The results achieved in the study of pig faces have gradually turned people’s attention to other animal face recognition, and other animal face recognition has begun to attract people’s attention. Yao, L. et al. proposed a cattle face recognition framework using a dataset containing about 50,000 annotated cattle face detection data and 18,000 cow recognition data. They proposed a hybrid detection and recognition model to improve the recognition performance of the method with 98.3% accuracy for detection, and 94.1% accuracy for cow face recognition [19]. Andersen, Pia Haubro et al. used two methods for horse face recognition. Preliminary results indicate that dynamics are essential for pain recognition and show that recurrent neural networks can classify experimental pain in horses better than human raters [20]. Zang, X.L. et al. introduced an algorithm for oblique image correction of cow faces. The algorithm used color image preprocessing and image binarization to separate the cow’s facial image from the background. It improved the computational speed by improving the integral projection function and reducing the image resolution. Finally, it improved the accuracy of the angle according to the symmetry of the cow’s face and corrected the cow’s facial image according to the angle. Experiments show that the algorithm has good computational speed and correction effect [21].

After studying biometric facial recognition comparison methods, research attention has also been extended to intelligent poultry farming systems and the study of chicken disease recognition. All these studies used deep learning [22,23,24,25,26,27,28]. Chicken counting and gesture research methods are also gradually being carried out. These studies provide the basis for the research on chicken face recognition and indicate the development direction.

Encouraged by the above research, this paper has the following main contributions:

1. A module of attention encoder was proposed to improve the accuracy of CNN models using the attention mechanism.
2. Improved the quality of the training set using various dataset enhancement methods.
3. Applied the attention encoder module to various mainstream CNN models for validation.
4. Several ablation experiments were implemented to discuss the factors affecting the performance of attention encoders.
5. An iOS-based chick day-age recognition application was developed.

The rest of this paper is organized as follows: (1) Section 2 describes basic information about CNNs and mainstream models. (2) Section 3 provides the details and preprocessing methods of the dataset. It also explains the structure of the attention model. (3) Section 4 gives the experimental environment and results. (4) Section 5 summarizes the whole article.

## 2. Related Works

In the 1980s, computers showed excellent processing ability in digital recognition because the multilayer perceptron model [29] was proposed. But due to the limitations of computing power, especially the processing power of CPU and storage resources, the size of the data that could be processed was small, the model expression ability was poor, and it usually could not handle complex picture problems. In 2006, Hinton et al. [30] proposed a layer-by-layer pre-training algorithm for network models, which enabled artificial neural networks with multiple hidden layers to have powerful feature learning capability by increasing the number of layers of artificial neural networks. They trained multilayer neural networks with small central layers to reconstruct high-dimensional input vectors, and effectively reduced the deep training difficulty of neural networks by encoding dimensionality reduction. In addition, other researchers have used support vector machines to overcome some of the difficulties encountered in training deep CNNs [31]. Afterward, the concept of deep learning and the rapid development of CNNs received much attention from researchers. In the early 21st century, many Internet technology companies such as Google and Microsoft invested a lot of human and material resources in developing and commercializing large-scale deep learning systems.

The convolutional model-a multilayer feedforward neural network model - its network structure is characterized by the use of a separate set of convolutional kernels in each layer. This structure helps to extract useful features from locally relevant data points. During the training process, CNNs learn through a backpropagation algorithm [32]. This backpropagation algorithm optimizes the objective function using a response-based human brain-like learning mechanism. The continued success of backpropagation algorithms and CNNs has led to a new phase of development in the field of artificial intelligence.

Deep architectures often perform better than shallow architectures when dealing with complex learning problems, especially after the LeNet convolutional neural network model [33] on the Minst dataset, related network models such as AlexNet [11], VGG [34], GoogLeNet [35], ResNet [36], and MobileNet [37] have emerged successively. They have been widely used in the fields of medical image processing and case segmentation.

### 2.1. Basic Structure of CNN

Modeled after biological neural networks, CNN uses a core weight-sharing network structure that allows them to scale the network model by varying the depth and width of the network. This chapter summarizes some representative components of popular deep neural network models.

#### 2.1.1. Convolutional Layer

CNN models have strong assumptions about natural images, namely statistical smoothness and local correlation. The convolution operation can effectively reduce the learning complexity of the network model with fewer connection and weight parameters, which makes it easier to train than a fully connected network of the same size. There are four common convolution operations: normal convolution [38], transposed convolution [39], dilated convolution [40], and deeply separable convolution [37].

Ordinary convolution is the process of sliding the convolution kernel over the image and finally completing the computation of gray values of all image pixels through a series of matrix operations. Transposed convolution implements the sampling operation in the reverse direction of ordinary convolution and is widely used in semantic segmentation [41], image recognition [42], etc. Dilated convolution, also known as hole convolution, injects holes into the convolution kernel to increase the perceptual field [43] of the model for better feature extraction. Dilated convolution has achieved better performance in tasks such as image recognition [44]; deeply separable convolution has also been extended for the lightweight network model MobileNet. Compared with the typical convolutional approach, it significantly reduces the number of parameters required for the network model operation. Most importantly, the depth-separable convolution separates channels and regions in the regular convolutional operation. The convolution method’s improvement alleviates the feature extraction problem to some extent.

#### 2.1.2. Activation Function Layer

**Figure 1:**
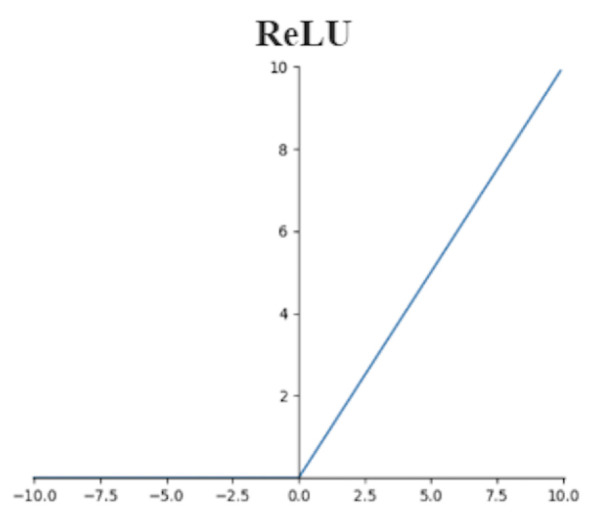
ReLU activation function.

The application of activation functions increases the nonlinearity of neural network models. The commonly used activation functions are Rectified Linear Unit (ReLU) [11], Randomized ReLU [45], Exponential Linear Unit (ELU) [46], and so on. ReLU is one of the most remarkable non-saturation activation functions, as shown in Figure 1, and its mathematical expression is as follows:

Although the discontinuity when ReLU is 0 may impair the backpropagation performance, it has been shown that ReLU is more effective than Sigmoid and tanh activation functions [47].

#### 2.1.3. Batch Normalization Layer

Gradient descent is a simple method used to train neural networks, but it requires artificial parameter selection, resulting in much of the researchers’ time consumption in uncertain tuning efforts. In 2015 the Google team proposed the idea of Batch Normalization (BN) [48]. This method allows researchers to choose a more significant learning rate, allowing the model to multiply in training while also giving the model fast convergence.

The BN layer avoids the problem of gradient dispersion and gradient explosion in addition to the data death of the ReLU activation function. It also reduces the difficulty of initializing the weights. Usually, for the data to be trained, the mean $\mu_{B}$ and variance of the current batch $\sigma_{B}^{2}$ of data should be calculated first. The output of BN is calculated according to the following equation:

(1) $$\hat{x}=\beta +\gamma \cdot \frac{x_{train}+\mu_{B}}{\sqrt{\sigma_{B}^{2}+\varepsilon }}$$

The parameter ε avoids division by zero and increases numerical stability; the learnable parameters β and γ are used to adjust the data to a reasonable distribution range. The statistics of the global training data $\mu_{r}$ and $\sigma_{r}^{2}$ are also updated iteratively according to Equations (2) and (3).

(2) $$\mu_{r}\leftarrow momentum\cdot \mu_{r}+\left(1-momentum\right)\cdot \mu_{B}$$

(3) $$\sigma_{r}^{2}\leftarrow momentum\cdot \sigma_{r}^{2}+\left(1-momentum\right)\cdot \sigma_{B}^{2}$$

Among them, momentum is a hyperparameter to be set to balance the update magnitude of $\mu_{r}$ and $\sigma_{r}^{2}$. The BN layer calculates the output $\hat{x}$ in the test phase according to Equation (4).

(4) $$\hat{x}=\beta +\gamma \cdot \frac{x_{text}+\mu_{r}}{\sqrt{\sigma_{r}^{2}+\varepsilon }}$$

Among them, $\mu_{r},\sigma_{r}^{2},\beta ,\gamma$ are obtained from the statistical or optimization results of the training phase and are used directly in the testing phase without updating these parameters.

#### 2.1.4. Pooling

The pooling layer is one of the standard components in current CNNs and has been named pooling since the AlexNet [11]. Pooling layers represent images by mimicking the human visual system to reduce the dimensionality of the data and use higher-level features.

In practice, the most commonly used pooling methods are max pooling, average pooling, spatial pyramid pooling, etc. In addition to reducing the model computation and information redundancy, the pooling operation also improves the model’s scale and rotation invariance to different degrees, effectively preventing overfitting. The improvements in various pooling methods also better achieve feature compression and extraction, significantly reducing the time required for model training.

### 2.2. Mainstream Models

Deep learning-based CNN can be used for image recognition and classification. This method automatically learns features from a large amount of data used to improve the performance of pattern recognition systems. Most of the current approaches of conventional image classification networks directly use common deep convolutional networks for direct image classification, such as AlexNet [11], VGG [34], GoogleNet [35], ResNet [36], MobileNet [37], and so on, which have been used in the ImageNet Large Scale Visual Recognition Challenge (ILSVRC) [49] to prove their application value.

## 3. Materials and Methods

### 3.1. Dataset Acquisition

**Figure 2:**
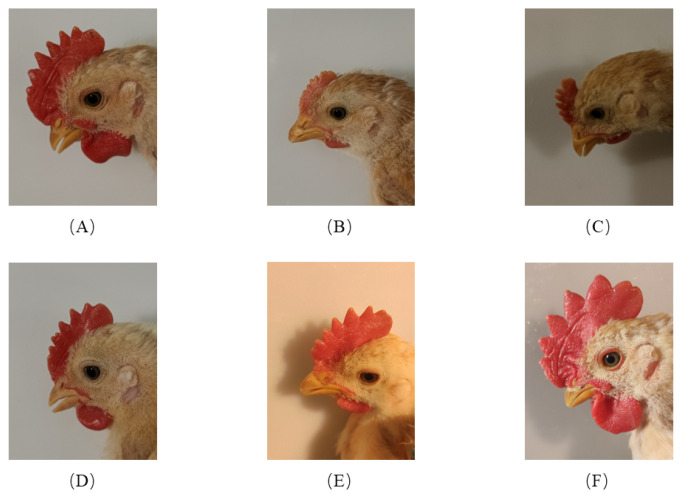
Illustration of dataset, (**A**)–(**F**) are images of chickens at different day-ages.

The dataset was collected by the Guangdong Academy of Agricultural Sciences on the fourteenth day after the chickens broke their shells (20 March 2022) by Canon 5D. The images’ resolution in this dataset is 6720×4480, as shown in Figure 2 and Table 2.

### 3.2. Dataset Augmentation

As shown in Table 2 and Figure 2 the dataset in our paper has following features:

1. The dataset contains many kinds of day-age chicken, some of which are very close in appearances;
2. Uneven distribution of samples in the dataset;
3. The scale of dataset is small, which makes deep learning training very difficult.

**Figure 3:**
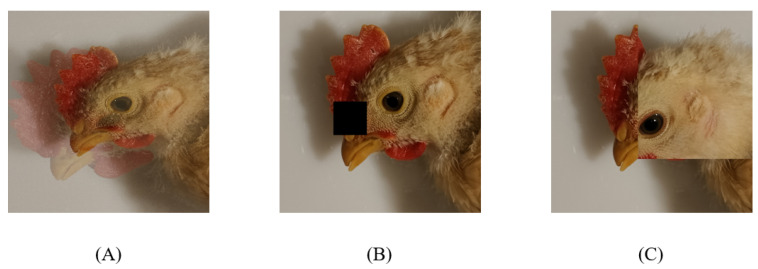
Illustration of different dataset augmentation methods. (**A**) Effect of using Mixup method; (**B**) Effect of using Cutout method; (**C**) Effect of using CutMix method.

In order to improve image quality and enhance model robustness, the following dataset enhancement methods are used. Their visualization effects are demonstrated in Figure 3:

1. Mixup [50]: Mix two random samples proportionally, and the classified results are assigned proportionally, as shown in Figure 3A;
2. Cutout [51]: Randomly cut out part of the sample and fill it with 0-pixel values. The classification result is unchanged, as shown in Figure 3B;
3. CutMix [52]: A portion of the region is cut off but not filled with 0 pixels, but randomly filled with the pixel values of the other data in the training set, as shown in Figure 3C. Moreover, the classification results are distributed in a certain proportion.

The difference between the above three kinds of data enhancement is: Cutout and CutMix is the difference between the pixel values of the filled area. Mixup and CutMix is the difference in the way of mixing two kinds of samples: Mixup interpolates the two images proportionally to mix the samples; CutMix is to mix the images by cutting part of the area and then patching it, so there will be no unnatural situation after image mixing. Using these enhancement methods has the following advantages:

1. No non-informative pixels appear during the training process, thus enabling more efficient training;
2. Retain the advantages of regional dropout, and it can focus on the non-discriminative parts of the target;
3. Add information about other samples to the region cut by asking the model to identify objects from a local view, which can further enhance the positioning capability of the model;
4. There is no unnatural image blending, which can improve the performance of model classification;
5. The training and reasoning costs remain the same.

### 3.3. Methods

**Figure 4:**
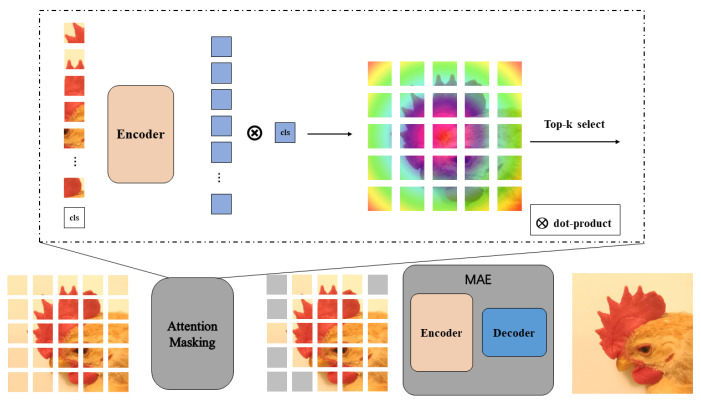
Illustration of our methods.

In this paper, we first use MAE to perform feature extraction experiments on the dataset, and the experimental results show that MAE can effectively reduce the dataset in this paper (the mask rate is set to 75%). After that, the features extracted by MAE in this paper are passed into a downstream classification model, such as the classical CNN model, for classification, and the overall process is shown in Figure 4.

#### 3.3.1. Attention Encoders

Give an unlabeled training set, attention encoders aim to learn encoders with parameters representing block-by-block binary masks with a patch size of 16 × 16 pixels.

At the same time, a decoder with parameters is trained to recover the original image from the potential embedding of the masked image, which denotes the reconstructed image. In this paper, the encoder and decoder were trained end-to-end, and the learning objective was the mean square error (MSE) between the reconstructed image and the original image in pixel space.

Experiments show that even compressing the model size of attention encoders can still achieve reasonably high performance. Therefore, to strike an ideal balance between speed and performance, we designed a compressed version of attention encoders and integrated it with ResNet-50 to perform downstream classification.

#### 3.3.2. Mask on Attention

In order to guide and enhance object perception, this paper utilized the inductive bias of object location in the masking strategy. Moreover, attention detection was used as a reasonable judgment to determine whether patches belong to foreground objects or not. The highly attentive patches were taken as input, and the remaining patches were removed. The encoder that was given the pre-trained can compute the attention graph for each input patch.

To fit the input format of the visual transformer, the input image was divided into non-overlapping patches, where (H,W) denotes the height and width of the image input image, *C* denotes the channel dimension, and *p* denotes the patch size. Recent studies have shown that a visual transformer trained without supervision can automatically learn object-related representations. Moreover, the attention graph of the CLS token can provide reliable foregrounding suggestions. The procedure for computing the attention on the image $patch_{i}$ is shown in Equation (5).

(5) $$Attn_{i}=q_{cls}\cdot k_{i},i\in \left\{0,1,\cdots ,p^{2}-1\right\}$$

#### 3.3.3. Augmentation by Attention

In this paper, the masked image was first acquired by the binary mask of attention. Then, the mask image was divided into non-overlapping patches, and the mask patch was discarded. The remaining visible patches were fed to the pre-trained encoder and decoder to generate the reconstructed images. The reconstructed image can be seen as an enhanced version that can be used for a variety of classification tasks. Note that once pre-trained, no further fine-tuning is required when testing on different datasets and tasks.

### 3.4. Baseline Models

#### 3.4.1. VGG

VGG convolutional neural network is a series of models proposed by Oxford Visual Geometry Group, a research team at the University of Oxford, in Large Scale Visual RecognitionChallenge in 2014. It includes VGG-11, VGG-13, VGG-16, VGG-19, etc. The main improvements of VGG are:

1. The VGG network model can be made 19 layers deep, which significantly increases the number of layers in the network.
2. The size of the convolutional kernels is reduced by using all 3 × 3 kernels whose stride is 1 and padding is 1. Compared with the 11 × 11, 5 × 5, and 3 × 3 kernels in AlexNet, it has fewer parameters and lower computational costs.
3. The VGG convolutional network model uses a combination of several small convolutional kernels (3 × 3), which is better than using one large convolutional kernel (5 × 5, 11 × 11). This is because using small-sized convolutional kernels reduces the number of parameters and provides the additional benefit of low computational complexity. It also verifies that performance can be improved by continuously deepening the network structure.

#### 3.4.2. GoogLeNet

GoogLeNet is a model proposed by the Google team at the Large Scale Visual Recognition Challenge in 2014. GoogLeNet and VGG both have relatively deep network layers. Although GoogLeNet has 22 layers, it has only 5 million parameters. AlexNet has 15 times more parameters than GoogLeNet, and VGG has three times more parameters than GoogLeNet. Therefore, GoogLeNet is a better choice for image classification when computer hardware resources are limited.

**Figure 5:**
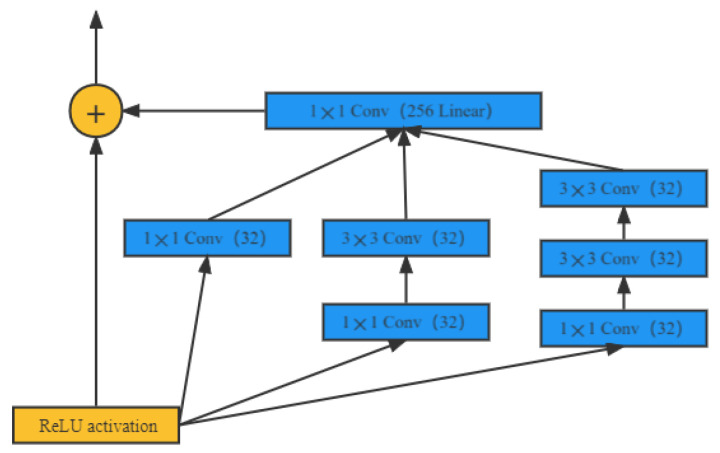
Illustration of inception.

The Google research team proposed the Inception (initial module) concept to construct the underlying neurons and build a network structure for sparse high-performance computing. As shown in Figure 5, in this CNN, one convolutional layer contains multiple convolutional operations of different sizes, which can generate dense data and ensure the efficient use of computational resources. After the original Inception V1 module, Google has successively proposed Inception V2, Inception V3, Inception V4, and other module structures, and its improvement has again made significant progress. In addition, it uses sparse connections to solve the problem of redundant information and to reduce the cost by omitting irrelevant feature maps. Finally, the GoogLeNet network model uses global average pooling to reduce the connection density instead of using a fully connected layer.

#### 3.4.3. ResNet

From the experience of VGG-19 and GoogLeNet models, the more layers of the network mean that the richer features can be extracted to different levels. In addition, the deeper the network, the more abstract and semantically informative features are extracted. However, it has been proved that the training effect of the deep network training model is worse as the number of layers of the network model increases. The ResNet model proposed by He et al. in 2015 solves the problem that the general network model has an increased error rate due to increasing the number of convolutional layers. The residual network is a way to improve the efficiency of information propagation by adding directly connected edges to the nonlinear convolutional layers. Suppose that in a deep network, a nonlinear unit (which can be one or more convolutional layers) F(x) is expected to approximate an objective function H(x) and split the objective function into two parts: the constant function and the residual function.

(6) H(x)=x+F(x)

**Figure 6:**
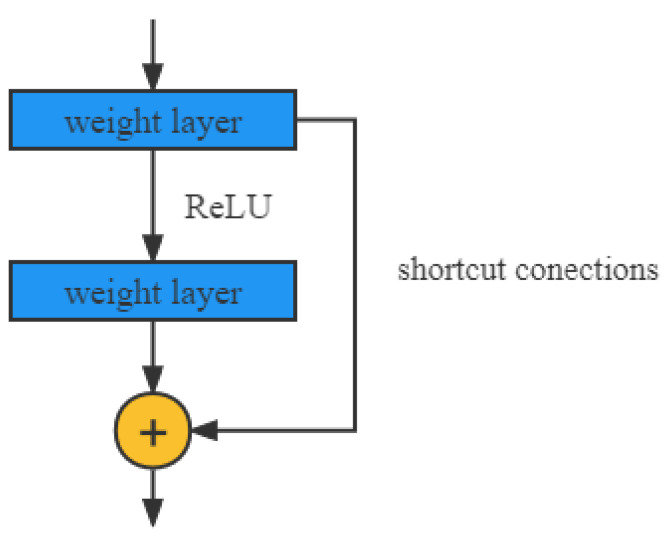
Illustration of shortcut connection.

Among them: H(x) is the residual module; *x* is the constant function; F(x) is the residual function. The principle is shown in Figure 6.

#### 3.4.4. MobileNet

**Figure 7:**
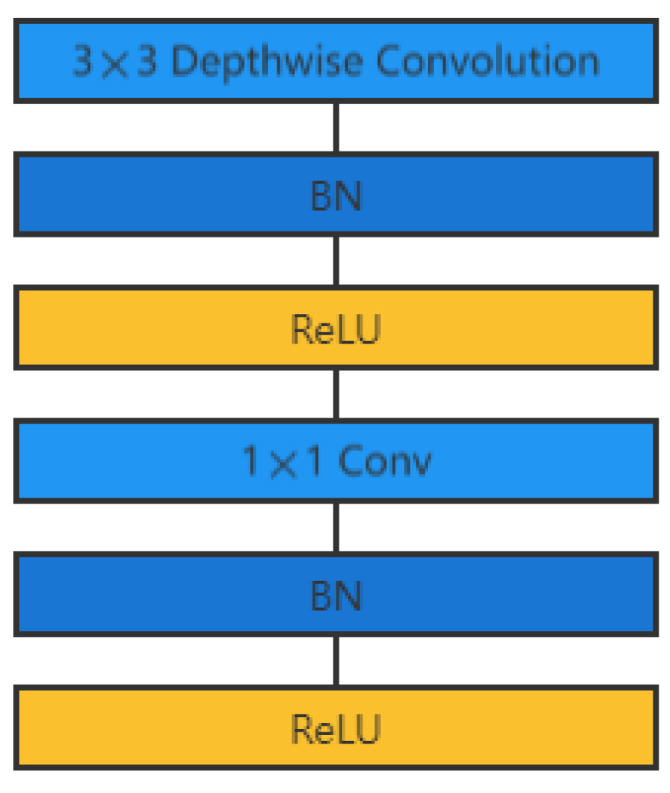
Illustration of deep separable convolution layer structure.

Embedded devices cannot use complex and large models, so it is vital to investigate small and efficient convolutional network models. The Google team proposed MobileNet [37] in 2017, which is a lightweight CNN focused on mobile or embedded devices. Subsequently, Google proposed MobileNetV2 in 2018 and MobileNet V3 in 2019. Compared with the traditional CNN, MobileNet V3 enables a significant reduction of model parameters at the expense of a small accuracy. The test results on ImageNet show that its accuracy is reduced by 0.9% compared with the VGG-16 model, but its model parameters are only 3.1% of those of the VGG-16 model. MobileNet uses depth-separable convolution layers, as shown in Figure 7, i.e., depthwise convolution is first performed on each channel of the feature map. Then, pointwise convolution is performed point by point to reduce the amount of computation and the number of model parameters.

## 4. Results and Discussion

### 4.1. Platform and Parameters

The test device is a desktop computer with a Core i9-10900k CPU and Nvidia RTX3080 GPU. In the training process, the experiments were run on Ubuntu 20.14, using the Python programming language, and the model implementation was based on the PyTorch framework. The number of learning rounds was set to 150, and the network was optimized using the stochastic gradient descent algorithm, where the initial learning rate is $1\times 10^{-5}$.

### 4.2. Experiment Results

The results of the experiment are shown in Table 3.

The model using ResNet-50 as the backbone achieves 95.2% accuracy, outperforming a range of automatic augmented search methods. Also, this paper compared the GPU hours of pre-training and pre-search. Moreover, once pre-trained, the model in this paper can be applied to multiple classification tasks without additional fine-tuning. CutMix and its variants can be used to obtain better results by introducing inter-sample regularization. This model can also be combined with CutMix to improve performance further.

To further investigate the accuracy of ResNet-50 on different day-age, we conducted experiments and obtained Table 4.

From the experimental results, it can be seen that the model is most accurate in identifying the day-age of the three periods-1–10, 10–15 and 15–20, and its accuracy rate is over 97%. The accuracy of the model for the three periods 21–24, 25–28 and 29–32 decreased, probably because the time steps of the last three periods were shorter and the changes of the facial features of chickens in this period were smaller.

We also tested the generalization performance of our model on several fine-grained classification datasets. For all experiments, 90 rounds of fine-tuning were performed on ResNet-50 from the official pre-training checkpoint provided by PyTorch. To ensure a fair comparison, we keep the hyperparameters identical during the experiments running the baseline and present models. The experimental results are shown in Table 5.

From the results in the above table, it can be seen that the model in this paper can effectively improve the performance of fine-grained classification.

### 4.3. Ablation Study

In order to investigate how the masking rate affects the model performance, this paper experimented with masking rates ranging from 20% to 80%. The experimental results are shown in Table 6.

The experimental results show that the pre-trained mini-Model achieves the best performance at a rate of 40%. This suggests that smaller models may not converge well at higher masking rates. However, a minimal masking rate can also make the pre-training task too easy, which may affect the generalization ability of the pre-trained mini-Model.

Therefore, in this paper, we conduct experiments for different model sizes, and the results are shown in Table 7.

The experimental results show that using a larger model as an enhancer brings higher classification accuracy for the same masking rate. This is because the larger model captures more accurate attention information and provides more robust regularization. However, the memory and speed costs of the large-Model are unaffordable. By tuning the masking rate, the mini-Model achieves better performance with a 6x speedup and 95% parameter reduction compared to the large model.

The Pretraining epoch is an important hyperparameter for self-supervised learning. For example, MoCo-v2 requires 800 epochs, and MAE requires 1600 epochs to converge with large models. Therefore, this paper investigated the model’s accuracy with different pretraining epochs. The result is shown in Table 8.

The experimental results show no significant difference in model performance when extending the pre-training epochs from 200 to 800, indicating that the 200 pre-training epochs are sufficient for the mini-Model.

### 4.4. Intelligent Chick Recognition System

#### 4.4.1. Wireless Collection System for Chicken Farms

**Figure 8:**
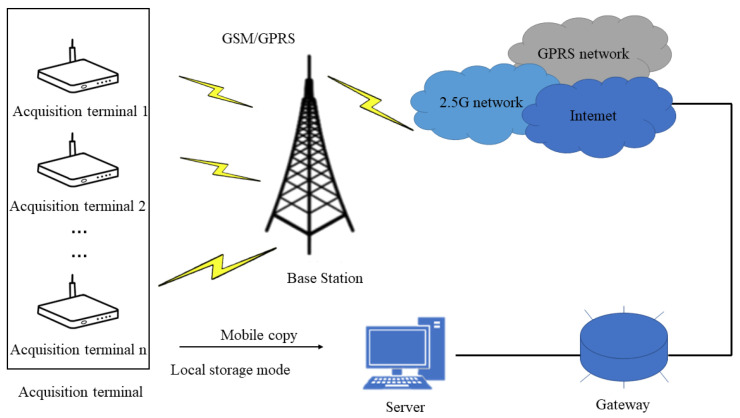
Illustration of wireless collection system for chicken farms.

Based on the existing system, this paper designed a broiler farm acquisition system based on wireless transmission technology, which includes three parts: the acquisition terminal, the wireless communication module, and the remote server, as shown in Figure 8.

Among them, the acquisition terminal is placed in the broiler farm to collect the broiler video signal regularly or in real-time and add a filtering algorithm to collect high-quality signals. The wireless communication module specifically contains GSM/GPRS communication module and WiFi communication module, which are used to establish the socket communication connection between the collection terminal and the remote server, and send the collected audio signals from the collection terminal to the remote server. After the remote server receives the signal, it stores it in the external memory so that the manager can further process the signal.

The system is currently deployed in Rizhao City, Shandong Province, China. This system helps chicken farm staff automatically detect the behavior of chickens.

#### 4.4.2. Application of Chick Day-age Recognition

**Figure 9:**
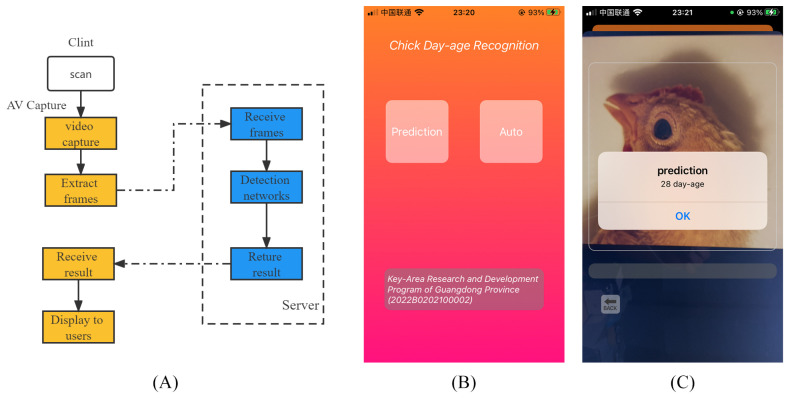
Illustration of chick day-age recognition application. (**A**) demonstrates the technical process of the application; (**B**) is a screenshot of the application; (**C**) is a screenshot of the application in use.

In addition, in order to apply the model in this paper to a practical scenario, we designed a chicken day-age recognition application based on iOS development techniques, as shown in Figure 9.

Figure 9A shows the technical process of the application, and Figure 9B is a screenshot of the application in use.

## 5. Conclusions

This paper proposed a compressed attention encoder structure to extract chicken face features in images. Various data augmentation methods were conducted to tackle the dataset’s insufficiency and imbalance and enhance the model’s generalizability. Compared with other mainstream CNN networks, the presented attention structure gained the most satisfactory performance when using the ResNet-50 as the backbone, with 140 GPU hours and 95.2% accuracy. Furthermore, we explored the scale and other hyperparameters’ effects on the model. By adjusting the masking rate, the mini-Model achieved the best performance with 95.8% accuracy, and it reduced parameters compared to the large model. Meanwhile, 200 pre-training epochs are sufficient for the mini-Model. Ultimately, we established a complete image acquisition system for chicken coops and a detection application configured for mobile devices to implement the model in practical production scenarios. This application can satisfactorily recognize the day-age of a chick, and it provides an excellent example for using artificial intelligence techniques to determine poultry’s day-age with high accuracy and speed.

Despite the contributions mentioned above in this paper, there are still some limitations that are worthwhile further exploring:

1. Although this paper was able to achieve a high average accuracy to achieve recognition at different day-ages, its recognition accuracy still needs to be improved in some specific time periods, such as 24-28 day-ages.
2. Utilizing the model in this paper when the number of datasets is not sufficient may lead to degradation of model performance.

These limitations will be the future work and research interests of authors in this paper, and other scientists.

## Figures and Tables

**Table 1:** The information about chickens in diverse classification standards.

Standards	Types
production status	egg-laying chickens
	laying chickens
	replacement chickens
	nesting chickens
type of production	egg-laying chickens
	breeding chickens
ages	new chickens (chickens in their first year of egg-laying)
	2-year-old chickens
	3-year-old chickens

**Table 2:** Distribution of different day-age data sets.

Day-Age	Number of Dataset
1-10	2819
10-15	1549
15-20	1821
21-24	983
25-28	840
29-32	975

**Table 3:** Experiment results on different models.

Method	GPU Hours	Mean Accuracy
VGG-13	450	91.9
VGG-16	450	93.8
VGG-19	450	92.3
ResNet-50	140	95.2
ResNet-101	140	93.8
GoogLeNet	575	94.3
MobileNet	280	93.9

**Table 4:** Experiment results on different day-age of ResNet50.

Day-Age	Accuracy
1-10	98.1
10-15	97.8
15-20	97.3
21-24	94.7
25-28	93.1
29-32	93.8

**Table 5:** Accuracy on small scale datasets.

Method	Accuracy
baseline (ResNet-50)	84.52
ours	86.71

**Table 6:** Accuracy on different mask rate.

Mask Rate	Accuracy
baseline	94.5
low	94.7
medium	95.5
High	94.5

**Table 7:** Comparison of different size of our model.

Method	Encoder	Decoder	Dimention	Mask-Rate	Parameters	Accuracy
mini-Model	4	2	480	0.75	16 M	95.1
mini-Model	4	2	480	0.5	16 M	95.8
base-Model	12	8	768	0.75	112 M	95.3
large-Model	12	8	1024	0.75	323 M	95.7

**Table 8:** Comparison of different pretraining epochs.

Epoches	Accuracy
100	95.2
200	95.7
400	95.7
800	95.6

## Data Availability

Not applicable.
